
JOURNAL INFORMATION
==============================
NLM Title Abbreviation: EFSA J
Journal ID: EFSA J
NLM Journal ID: 101642076
Journal ID: EFS2

EFSA Journal

EISSN: 1831-4732
Publisher: Wiley

ARTICLE INFORMATION
==============================
PMCID: PMC10731992
PMID: 38125971
DOI: 10.2903/j.efsa.2023.p211203
Article ID: EFS2P211203
Article version: 1
Subjects: Plain Language Summary, Plain Language Summary

Plain Language Summary on the Re‐evaluation of erythritol (E 968) as a food additive

Electronic publication date: 2023 Dec 20
Publication date: 2023 Dec
Volume: 21
Issue: 12
Electronic Location ID: p211203
Copyright: © 2023 European Food Safety Authority. EFSA Journal published by Wiley‐VCH GmbH on behalf of European Food Safety Authority.
License: This is an open access article under the terms of the http://creativecommons.org/licenses/by/4.0/ License, which permits use, distribution and reproduction in any medium, provided the original work is properly cited.
License URL: https://creativecommons.org/licenses/by/4.0/

Figure count: 0
Table count: 0
Page count: 3

source-schema-version-number: 2.0
cover-date: December 2023
details-of-publishers-convertor: Converter:WILEY_ML3GV2_TO_JATSPMC version:6.3.6 mode:remove_FC converted:20.12.2023

Note: This is a plain language summary of EFSA’s scientific opinion 8430 which is available at https://efsa.onlinelibrary.wiley.com/doi/10.2903/j.efsa.2023.8430

==============================

REFERENCE

Re‐evaluation of erythritol (E 968) as a food additive. DOI: 10.2903/j.efsa.2023.8430
